# Conservation of gene co-expression networks and neuropsychiatric-neurological disease gene programs between mouse and human frontal cortex at single-cell resolution

**DOI:** 10.64898/2026.10.05.756860

**Published:** 2026-10-06

**Authors:** Qian-Quan Sun, Tianyu Cao, Chunzhao Zhang

## Abstract

Mouse models are widely used in translational neuroscience, but whether a gene has the same partners in mouse and human prefrontal cortex (PFC) is unknown. The genes whose expression rises and falls with a given gene across samples (its co-expression partners) indicate the molecular program to which it belongs. We compared the co-expression of 13,214 genes between mouse medial PFC and human dorsolateral PFC in 246,211 single-cell and single-nucleus RNA-sequencing profiles. These two regions are not homologous, so part of any measured difference may reflect region and not species. The partners of single genes were only modestly similar in the two species, whereas groups of co-expressed genes (modules) were strongly preserved, and the higher conservation of disease genes was largely accounted for by general gene properties. We report five findings. First, the median similarity of partners for single genes was 0.169 (0, unrelated; 1, identical). This median was below that of every comparison among human datasets but within the range of comparisons among mouse datasets, and correction for measurement noise raised it to 0.349 at most. Second, all four mouse modules and four of five human modules were strongly preserved in the other species. Third, neuropsychiatric disease genes were modestly more conserved than other genes, but no more than control genes matched for mutational constraint and expression. None of the 14 disease genes had fewer cross-species partners than connectivity-matched comparison genes. Fifth, in an independent human dataset, four of five human modules defined on the 5,000 most variable genes recurred. We also identified two sources of error: analyzing only the most variable genes excludes most disease genes, and networks built from few samples, as for glial cells, underestimate the similarity between species. Scores and code are released for cross-species studies.

## Full Text Availability

The license terms selected by the author(s) for this preprint version do not permit archiving in PMC. The full text is available from the preprint server.

